# Quarterly Summary of the Transactions of the College of Physicians of Philadelphia

**Published:** 1843-06-10

**Authors:** 


*Quarterly Summary of the Transactions of the College of Physicians of Philadelphia. February, March, and April*, 1843. 8vo. pp. 34.

The present number of the Transactions contains the Annual Report on the Diseases of Women, by Dr. Warrington; and the Annual Report on the Diseases of Children, by Dr. Condie. Dr. Warrington, in his report, has collected the various observations on his subject which have appeared in the periodical publications, and regrets that "he has been enabled to comprise so few facts of either a novel or particularly interesting character." The regret expressed with regard to the erroneous nature of the system of physical education of females, and the opinion that, until a rational improvement in this respect be effected, chlorosis will continue to prevail, will be generally shared by practitioners. The success of Electricity in Amennorrhœa, which Dr. Golding Bird, of Guy's Hospital, has obtained, is alluded to. In all these cases, if we recollect aright, the patients were subjected to a general treatment at the same time. It would not be difficult to assign the relative parts played by the exercise, regimen and tonics, and the electricity. Much interesting detail is presented with regard to the sore mouth peculiar to nursing women.

The paper of Dr. Condie contains a concise but full summary of the progress of the department confided to him, intermingled with many judicious observations.

---

M. Pitschaft, of Baden, asserts that he has cured incontinence of urine in patients of either sex by small doses of strychnine. To insure success, however, it is necessary that the bowels should be cleared before commencing the use of this remedy.—*London Lancet.*

